# Inhibition of tumor growth and histopathological changes following treatment with a chemokine receptor CXCR4 antagonist in a prostate cancer xenograft model

**DOI:** 10.3892/ol.2013.1515

**Published:** 2013-08-06

**Authors:** KANG SU CHO, SO JUNG YOON, JOO YONG LEE, NAM HOON CHO, YOUNG DEUK CHOI, YUN SEOB SONG, SUNG JOON HONG

**Affiliations:** 1 Department of Urology and Urological Science Institute, Severance Hospital, Yonsei University College of Medicine, Seoul 120-752, Republic of Korea; 2 Department of Pathology, Severance Hospital, Yonsei University College of Medicine, Seoul 120-752, Republic of Korea; 3 Department of Urology, Soonchunhyang University Hospital, Soonchunhyang University College of Medicine, Seoul 140-743, Republic of Korea

**Keywords:** prostatic neoplasms, receptors, CXCR4, chemokine, CXCL12

## Abstract

The stromal derived factor-1 (SDF-1)/CXCR4 axis is associated with tumor aggressiveness and metastasis in prostate cancer. The present study aimed to explore the potential therapeutic effects of a CXCR4 antagonist in prostate cancer. The effect of SDF-1 and a CXCR4-specific antagonist, AMD3100, on human prostate cancer PC-3 cell proliferation and protein kinase B (Akt) signaling was assessed. Moreover, a PC-3 tumor xenograft model was used to evaluate the effect of AMD3100 on tumor growth and to identify the histopathological changes and immunohistochemical differences between AMD3100-treated and untreated groups. Cell proliferation was not significantly affected by SDF-1 or AMD3100 treatment *in vitro*. Western blot analysis revealed that SDF-1 stimulation enhanced the expression of phosphorylated Akt in the PC-3 cells, but that the SDF-1-induced expression of phosphorylated Akt was abrogated in the AMD3100-treated PC-3 cells. In the PC-3 tumor xenograft model, AMD3100 significantly inhibited tumor growth, while AMD3100-treated PC-3 tumors had lower levels of microvessel formation and a lower immunoreactivity for the proliferation marker Ki-67 and the anti-apoptotic marker Bcl-2 compared to control tumors *in vivo*. The CXCR4-specific antagonist inhibits SDF-1-induced CXCR4/Akt signal transduction, and effectively suppresses tumor growth in the PC-3 xenograft model. The present study indicates that CXCR4 targeting may represent a novel strategy for the treatment of castration-resistant prostate cancer (CRPC).

## Introduction

Radical surgery or radiotherapy may be curative therapies for patients with localized prostate cancer, but there are no effective treatment modalities for the management of advanced prostate cancer. Androgen deprivation treatment is the most effective systemic approach for patients with metastatic disease. Although 80–90% of patients initially respond favorably to this treatment, they eventually become unresponsive to androgen deprivation and develop castration-resistant prostate cancer (CRPC) (1,2). Currently, the combination of docetaxel and prednisone is considered the standard first-line therapy for CRPC, but the survival gain from docetaxel chemotherapy is limited and unsatisfactory (3).

Stromal derived factor-1 (SDF-1) is a member of the CXC subfamily of chemokines that interacts with the 7 transmembrane G-protein-coupled receptor CXCR4 (4–6). CXCR4 expression has been reported in at least 23 epithelial, mesenchymal and hematopoietic cancers, indicating the importance of this ligand/receptor axis in tumor aggressiveness and metastasis (7–9). The role of the SDF-1/CXCR4 axis in prostate cancer has been experimentally demonstrated. It is known that CXCR4 mRNA and protein are expressed in prostate cancer cell lines, including LNCaP, PC-3 and DU145, and in human prostate samples (10). Akashi *et al*(11) demonstrated that patients with a high level of CXCR4 expression in the tumor had worse cancer-specific survival rates than patients with a low level of expression. Darash-Yahana *et al*(12) reported that subcutaneous xenografts of prostate tumors that overexpressed CXCR4 in nude mice were 2- to 3-fold larger in volume and weight compared to the controls. Moreover, the blood vessel density, invasiveness of the tumors and metastasis to the lymph nodes and lungs were significantly increased in these tumors. In the present study, the potential therapeutic effects of the CXCR4 antagonist were explored in a prostate cancer xenograft model.

## Materials and methods

### Cell culture and reagents

Human prostate cancer PC-3 cells, which are representative of CRPC cells, were obtained from the Korea Cell Line Bank (Seoul, Korea). These cells were maintained in RPMI-1640 supplemented with 10% fetal bovine serum (FBS), 1% penicillin-streptomycin and 1% L-glutamine. All cells were grown in a humidified incubator at 37°C and 5% CO_2_. SDF-1 (R&D Systems, Minneapolis, MN, USA) was used as a specific ligand for CXCR4, and the bicyclam derivative, AMD3100 (Sigma-Aldrich, St. Louis, MO, USA), was used as a CXCR4-specific antagonist. In certain experiments, the cells were pretreated with filipin (Sigma-Aldrich) to deplete membrane cholesterol (13).

### In vitro proliferation assay

The PC-3 cells were seeded at a density of 2×10^3^ cells/well into 96-well plates in culture medium containing 10% FBS. After 24 h, the cells were washed and cultured with serum-free medium alone (control) or with medium containing SDF-1 or AMD3100 at various concentrations. After 72 h, the number of viable cells was counted using a CellTiter 96^®^ AQueous One Solution Cell Proliferation Assay (Promega, Madison, WI, USA) according to the manufacturer’s instructions. This assay is based on the ability of viable cells to bioreduce 3-(4,5-dimethylthiazol-2-yl)-5-(3-carboxymethoxyphenyl)-2-(4-sulfonyl)-2H-tetrazolium to formazan in the presence of phenazine methosulfate, an electron-coupling reagent. Formazan production was quantified by measuring absorbance at 490 nm, which is directly proportional to the number of living cells.

### Western blot analysis

Prostate cancer cells were cultured to subconfluence (80–90%), washed and incubated in serum-free media for 12 h. SDF-1 stimulation was performed with 0–200 ng/ml SDF-1 for various lengths of time. In certain experiments, 1 μg/ml AMD3100 or 1 μg/ml filipin was pre-incubated with the cells. The cells were lysed with radio-immunoprecipitation assay (RIPA) lysis buffer consisting of 50 mM HEPES (pH 7.6; USB, Cleveland, OH, USA), 150 mM NaCl (Sigma-Aldrich), 1% NP-40, 10 ml/ml phenylmethylsulfonyl fluoride (both Amresco, Solon, OH, USA) and 10 ml/ml aprotinin (Sigma-Aldrich), and cleared by centrifugation at 12,000 × g. The protein concentration was determined using a detergent compatible protein assay kit (Bio-Rad Laboratories, Hercules, CA, USA). Equal amounts of cell lysates were separated by 10–12% sodium dodecyl sulfate-polyacrylamide gel electrophoresis and transferred to nitrocellulose membranes. The membranes were blocked for 1 h at room temperature in 5% skimmed dried milk and incubated for 2 h at room temperature with anti-protein kinase B (Akt)1 (1:1,000), anti-p-Akt1/2/3 (1:1,000) or β-actin (1:2,000) antibodies (all from Santa Cruz Biotechnology, Inc., Santa Cruz, CA, USA). The membranes were incubated with horseradish peroxidase (HRP)-conjugated goat anti-rabbit immunoglobulin G (IgG) or goat anti-mouse IgG for 1 h at room temperature. Protein signals were detected by chemiluminescence with ECL detection reagents (Amersham Biosciences, Piscataway, NJ, USA).

### Xenografts in nude mice

All the protocols for the animal studies were reviewed and approved by the Institutional Animal Care and Use Committee at Yonsei University College of Medicine. Six-week-old male nude mice (BALB/c) were obtained from Japan SLC (Hamamatsu, Japan). The PC-3 cells (5×10^6^ cells/mouse) were injected subcutaneously into the right dorsal region. When the tumors measured 40 mm^3^, the mice were randomized to receive AMD3100 (n=6; 3 mg/kg) or vehicle alone (n=6; phosphate-buffered saline) by intraperitoneal injection for 5 consecutive days/week for 4 weeks. Tumor diameters were measured at regular intervals using calipers. The tumor volume (V) was calculated using the following formula: V = A × B^2^/2 (A, axial diameter; B, rotational diameter). Tumors were excised and fixed overnight in neutral-buffered formalin and processed by routine methods.

### Immunohistochemistry

Immunohistochemical staining was performed using the mouse anti-human Ki-67 monoclonal antibody (1:50), the mouse anti-human Bcl-2 antibody (prediluted form; both from Dako, Carpinteria, CA, USA) and the rabbit anti-mouse CD34 monoclonal antibody (1:100; BD Pharmingen, San Jose, CA, USA) in a xenograft tissue experiment. In brief, formalin-fixed, paraffin-embedded 4-μm specimens were deparaffinized and rehydrated. The sections were treated with 2% hydrogen peroxide to inactivate endogenous peroxidase, and non-specific binding was blocked by treatment with the blocking reagent. The primary antibody was applied to each section for 1 h at 37°C, and the appropriate HRP-conjugated secondary antibody was applied at a dilution of 1:100 for 1 h at room temperature. Immunoreactivity was subsequently detected using a 3,3′-diaminobenzidine system (Vector Laboratories, Burlingame, CA, USA). Nuclei were counterstained using Meyer’s hematoxylin.

### Data analysis

Statistical comparisons were performed among groups using Student’s t-test or the Mann-Whitney U test. P<0.05 was considered to indicate a statistically significant difference. The Statistical Package for Social Sciences (SPSS), version 12.0 for Windows, was used for the statistical analysis. Data are expressed as the mean ± standard deviation. All *in vitro* experiments were repeated with triplicate or quadruplicate samples and similar results were obtained across all trials.

## Results

### Effect of SDF-1 and a CXCR4 antagonist on proliferation of PC-3 cells

**Figure 1:**
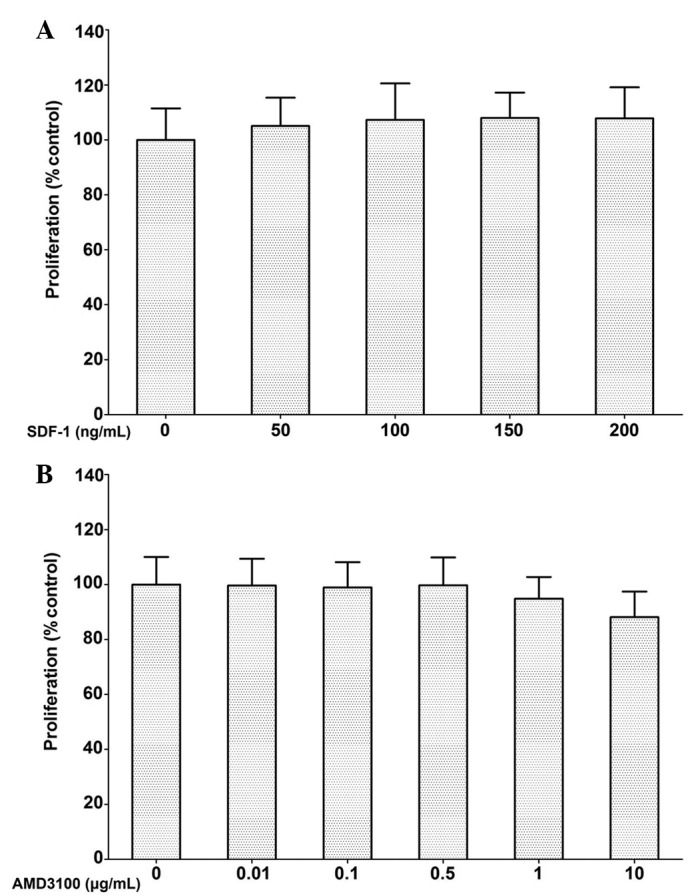
Effects of (A) stromal derived factor-1 (SDF-1) and (B) a CXCR4 antagonist on human prostate cancer PC-3 cell proliferation. Points, mean; bar, standard deviation. There was no statistically significant difference among groups. SDF-1, stromal derived factor-1.

Subsequent to incubation for 72 h, PC-3 cell proliferation was not significantly affected by SDF-1 at concentrations ranging between 50 and 200 ng/ml. AMD3100 also showed no inhibitory effect on PC-3 cell proliferation at concentrations ≤0.5 μg/ml. Although cell proliferation was marginally suppressed at 1 and 10 μg/ml, the effect was not statistically significant (Fig. 1).

### Effect of a CXCR4 antagonist on SDF-1/CXCR4-mediated Akt signaling in PC-3 cells

**Figure 2:**
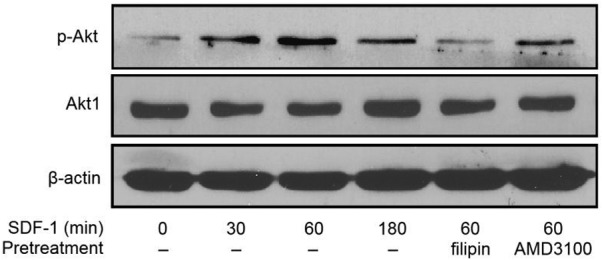
Effects of a CXCR4 antagonist and cholesterol depleting agent on stromal derived factor-1 (SDF-1)/CXCR4-mediated protein kinase B (Akt) signaling in human prostate cancer PC-3 cells. Serum-starved PC-3 cells were stimulated by 200 ng/ml SDF-1 for 0, 30, 60 and 180 min. In certain cases, serum-starved PC-3 cells were pretreated with filipin or AMD3100 prior to SDF-1 stimulation. SDF-1, stromal derived factor-1.

Following SDF-1 stimulation, the expression of phosphorylated Akt was enhanced in the PC-3 cells at 30, 60 and 180 min compared with the control. The highest levels of Akt phosphorylation following SDF-1 stimulation were observed between 30 and 60 min. The expression of pAkt induced by SDF-1 was marginally increased in the AMD3100-treated PC-3 cells compared to the controls, but the levels did not reach those expressed in the PC-3 cells stimulated by SDF-1 alone. Examination of the total Akt levels demonstrated that almost all of the conditions resulted in similar levels of Akt (Fig. 2). These results indicate that the CXCR4-specific antagonist AMD3100 abrogates SDF-1-induced pAkt activation.

### Blocking CXCR4 suppresses the tumor growth of prostate cancer in a xenograft mouse model

**Figure 3:**
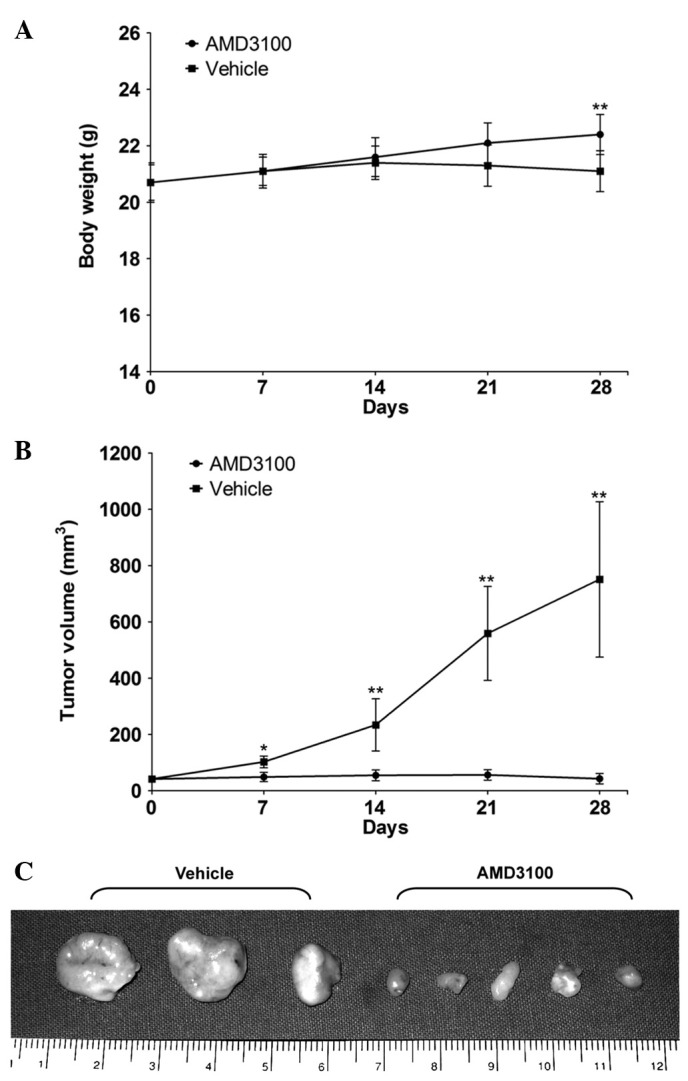
Effects of a CXCR4 antagonist on human prostate cancer PC-3 tumors *in vivo*. (A) Body weights and (B) tumor volumes of vehicle-treated (n=6) and CXCR4 antagonist-treated (n=6) nude mice are shown. The body weights and tumor volumes of the mice were measured at days 7, 14, 21 and 28. Points, mean; bar, standard deviation; ^*^P<0.05 and ^**^P<0.01, vs. vehicle. (C) Tumors were excised and images were captured, and the representative examples of PC-3 tumors are shown.

The tumor volumes of the AMD3100-treated and control groups were 49.0±16.7 and 102.5±20.9 mm^3^ on day 7 post-treatment, 54.9±19.4 and 234.0±92.9 mm^3^ on day 14, 56.3±18.5 and 559.9±167.8 mm^3^ on day 21 and 42.9±18.4 and 751.9±276.4 mm^3^ on day 28, respectively (Fig. 3). These data indicate that the CXCR4 antagonist used here delayed tumor growth at an early stage of tumor development. There was no difference in the body weight between the 2 groups until day 21, however, the body weight on day 28 was 22.4±0.7 g in the AMD3100-treated group and 21.1±0.7 g in the control group, and this difference was significant (Fig. 3).

### Histopathological examination of PC-3 xenograft tumors following CXCR4 antagonist treatment

**Figure 4:**
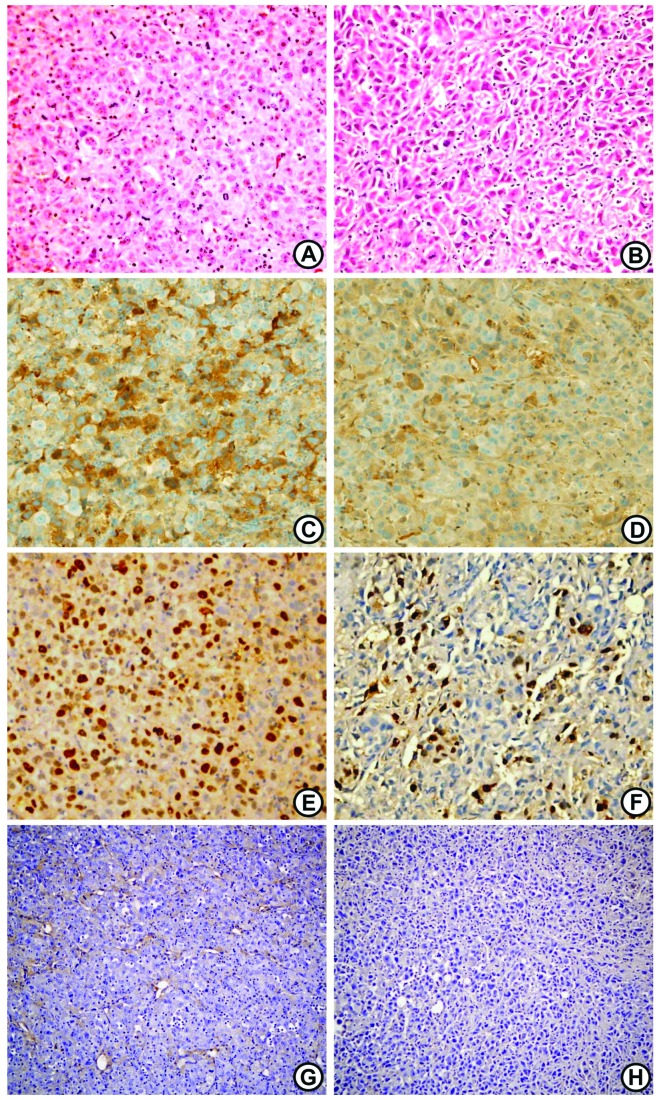
Histopathological examination of human prostate cancer PC-3 xenograft tumors following treatment with a vehicle or CXCR4 antagonist. Hematoxylin and eosin (HE) staining in (A) vehicle-treated and (B) CXCR4 antagonist-treated tumors. Immunohistochemistry of Bcl-2 in (C) vehicle-treated and (D) CXCR4 antagonist-treated tumors. Ki-67 in (E) vehicle-treated and (F) CXCR4 antagonist-treated tumors. CD34 in (G) vehicle-treated and (H) CXCR4 antagonist-treated tumors. (C and D) Immunohistochemistry for Bcl-2 expression showed brownish cytoplasmic staining. (E and F) For Ki-67, dark red-brownish nuclear staining was regarded as positivity. (G and H) There was a marked reduction in microvessel formation in the tumors of the CXCR4 antagonist-treated mice compared with those of the control group. Original magnifications (A–F) ×200; (G and H) ×100.

Hematoxylin and eosin (HE) staining revealed a definite histological change in the PC-3 xenograft tumors following treatment with the CXCR4 antagonist (Fig. 4). CXCR4 antagonist-treated tumors were characterized by their spindle cell shapes compared with the control tumors, as well as their enlarged, pleomorphic and hyperchromatic nuclei. Immunohistochemistry for Bcl-2 expression showed brownish cytoplasmic staining in the two groups, but Bcl-2 immunostaining was more predominant and more frequently observed in the control tumors (Fig. 4). The immunohistochemistry for CD34 was examined on the primary tumor tissue sections to determine whether the suppression of primary tumor growth was a result of an antiangiogenic effect (i.e., the inhibition of microvessel formation) of the CXCR4 antagonist. A marked reduction in microvessel formation was observed in the tumors of the CXCR4 antagonist-treated mice (Fig. 4).

**Figure 5:**
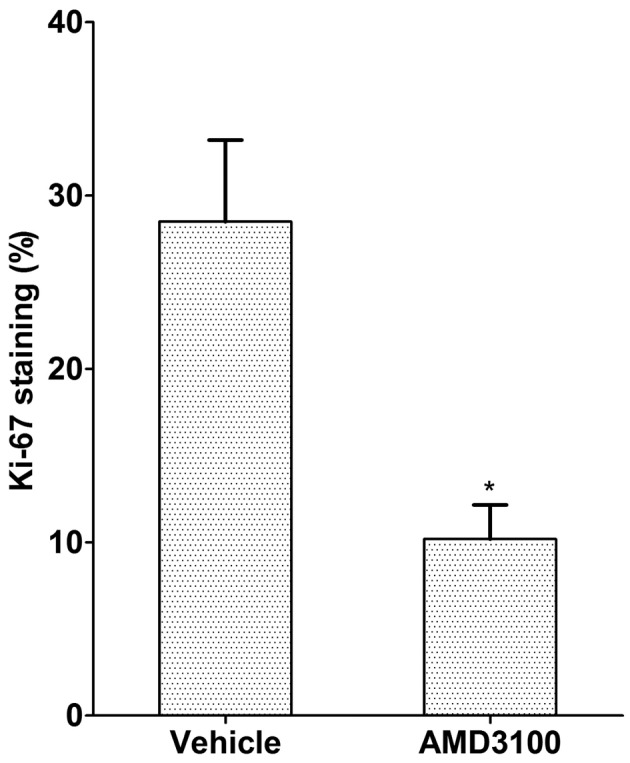
Ki-67 staining index of human prostate cancer PC-3 xenograft tumors following treatment with vehicle or CXCR4 antagonist. Points, mean; bar, standard deviation; ^*^P<0.05 vs. vehicle.

Ki-67 was used as an estimator of tumor aggressiveness, with dark red-brown nuclear staining regarded as positivity for Ki-67. The Ki-67 staining index was significantly higher in the control tumors compared to the CXCR4 antagonist-treated tumors (10.2±1.9 vs. 28.5±4.7%, respectively; P<0.05; Figs. 4 and 5).

## Discussion

The present study observed that a CXCR4-specific antagonist effectively inhibited CXCR4/Akt signal transduction in PC-3 cells, as well as tumor growth in nude mice challenged with PC-3 cells. This indicates that CXCR4 targeting may represent a novel, effective strategy for the treatment of human prostate cancer.

It is well known that the binding of chemokines to their G protein-linked receptors on target cells leads to a series of signal transduction events involving the generation of inositol 1,4,5-trisphosphate and cyclic adenosine monophosphate-dependent protein kinase, the activation of phosphatidylinositol 3-kinase (PI3K), the phosphorylation of protein kinase B (Akt) and extracellular signal-regulated kinase (ERK), the elevation of components of focal adhesion complexes and the activation of protein kinase C (14). SDF-1 binding to CXCR4 generates various signaling mechanisms affecting the regulation of angiogenesis, the activation of cell invasion, the promotion of cell growth, the inhibition of apoptosis, and notably, is important in metastasis (15–20). In a previous study of prostate cancer, differential activation of the ERK and PI3K/Akt pathways resulted in differential secretion of interleukin (IL)-6, IL-8, tissue inhibitors of matrix metalloproteinase-2 (MMP-2) and vascular endothelial cell growth factor, which affected the ability of the cancer cells to induce angiogenesis (15). Exogenous SDF-1 induces Akt phosphorylation in PC-3 cells, which is independent of PI3K and indispensable for MMP-9 secretion, migration and invasion (16). SDF-1 induction enhances various MMPs (MMP-1, -2, -3, -9, -11, -13 and -14) in PC-3 cells (17). It has also been reported that the SDF-1-induced expression of CXCR4 in PC-3 cells is dependent on the mitogen-activated protein kinase (MEK)/ERK signaling cascade and on nuclear factor-κB (NF-κB) activation, which enhances endothelial adhesion and transendothelial migration (18). An immunohistochemical study of human samples demonstrated that high NF-κB expression was associated with CXCR4 expression and that they are co-expressed in approximately one-third of patients with clinically localized prostate cancer (19). Wang *et al*(20) also showed that CXCR4 plays a significant role in prostate cancer metastasis through the upregulation of vascular endothelial growth factor (VEGF).

The present study showed that SDF-1 has no direct effects on PC-3 cell proliferation. These findings are in accordance with results from previous studies of other types of cells, showing that SDF-1 has no proliferative effect on glioma (U251n), cholangiocarcinoma (RMCCAI and KKU100), testicular germ cell tumor (TCAM2), rhabdomyosarcoma and pancreatic cancer cells (21–25). Notably, Sun *et al*(10) also showed that recombinant SDF-1 does not alter the growth rate of PC-3 cells under various conditions. However, an antibody to SDF-1 significantly decreased the number of PC-3 cells, which indicated that SDF-1 derived from the PC-3 cells themselves acts in an autocrine fashion to stimulate growth. Previous studies have demonstrated that SDF-1 stimulates the proliferation of small cell lung cancer cells (NCI-H69) in the presence of serum and colorectal cancer (SW480) and epithelial ovarian cancer (ES-2) cells in the absence of serum (26–28). In addition, antisense CXCR4 overexpression in glioblastoma cells has been shown to cause the inhibition of cell proliferation, indicating that the SDF-1/CXCR4 system is also involved in cell proliferation in glioblastoma cell lines (29,30). These differences may be due to different culture systems or target cells.

The effects of AMD3100 on the viability of tumor cells are controversial. In the present study, AMD3100 did not significantly affect the viability of the PC-3 cells. Glioma (U251n), testicular germ cell tumor (TCAM2), epithelial ovarian cancer (ES-2) and oral squamous carcinoma (B88-SDF-1) cells have also been shown to be insensitive to AMD3100 (24,25,28,31). The enhancing effect of SDF-1 on cell proliferation was markedly inhibited by AMD3100 treatment in colorectal cancer cells (SW480), but AMD3100 alone did not significantly affect cell proliferation when compared with the results observed in the SDF-1 unstimulated group, which indicated that there was no autocrine growth stimulatory loop in the cell line (27).

Although AMD3100 did not affect the viability of the PC-3 cells in the present study, it inhibited SDF-1-induced Akt activation. The Akt pathway is an important signaling pathway in prostate cancer (32,33). Akt is a serine-threonine kinase and its phosphorylation is linked to mitogenic signals. In addition to its role in survival, Akt participates in a number of intracellular pathways, including the integration of proliferation and differentiation signals that mediate migration and angiogenesis (16,32). To determine whether a CXCR4-specific antagonist inhibits SDF-1/CXCR4-mediated Akt phosphorylation, in the present study, the PC-3 cells were pretreated with AMD3100. The phosphorylation of Akt in the AMD3100 pretreated cells was significantly lower than in the untreated cells and was similar to that observed in studies of cholangiocarcinoma cells (21). It has also been reported that in SDF-1-stimulated activation of ERK1/2 and Akt, rapid responses to SDF-1 are attenuated by AMD3100 in medulloblastoma and glioblastoma cells (Daoy and U87) (34).

The present results clearly demonstrate the inhibitory effects of a CXCR4-specific antagonist on PC-3 tumor growth in nude mice, although AMD3100 treatment had no direct effect on the proliferation of PC-3 cells *in vitro*. Previous studies have investigated the effects of CXCR4 antagonism on tumor growth and metastasis in other xenograft models. Such studies have observed that AMD3100 effectively inhibits anaplastic thyroid carcinoma tumor growth (35). Intraperitoneal treatment with AMD3100 has been shown to result in reduced dissemination in nude mice inoculated with epithelial ovarian cancer cells (ES-2) (28). In an oral squamous cell carcinoma xenograft model, AMD3100 significantly inhibited lung metastasis of the SDF-1 transfectant, ameliorated body weight loss and improved the survival rates of tumor-bearing nude mice (31). Another anti-CXCR4 treatment, TN14003, has been shown to suppress primary tumor growth by inhibiting tumor angiogenesis and preventing lung metastasis of squamous cell carcinoma of the head and neck in animal models (36). The present study also showed that there was a marked reduction in microvessel formation in CXCR4 antagonist-treated tumors compared with tumors in the control group.

These marked differences in the biological effects of CXCR4 inhibition observed in animals and in cell culture may be explained by the fact that SDF-1 acts at multiple levels in the tumor microenvironment. Tumor stromal cells, including fibroblasts and bone marrow-derived cells, express high levels of SDF-1, which may directly enhance the growth of epithelial tumor cells and recruit endothelial progenitors, thus favoring angiogenesis (37). It is believed that chronic treatment with AMD3100 efficiently blocks SDF-1-mediated vasculogenesis. Accordingly, the suppression of tumor growth in treated mice may be explained by the inhibition of CXCR4^+^ tumor cell proliferation and the diminishing recruitment of CXCR4^+^ angiogenic cells. The present study observed a strong inhibition of PC-3 tumor growth following AMD3100 treatment, but AMD3100 did not induce a complete regression of the tumors. Therefore, we hypothesize that combined treatment with AMD3100 and antineoplastic agents, such as platinum or taxanes, is a promising strategy.

No toxic effects of AMD3100 were detected in the animal model of the present study, but the appropriate therapeutic approach for antagonizing CXCR4 remains unclear, as long-term sustained dosing of AMD3100 results in some toxicity (38). A previous study showed that the sustained dosing of AMD3100 over a 10-day period was associated with mild toxicities. Reflecting the effects of AMD3100 on bone marrow function, an elevation in white blood cell count was evident throughout an 18-day follow-up period following cessation of AMD3100 treatment (38). For these reasons, further studies aimed at understanding the effects of the long-term administration of CXCR4 inhibitors must be pursued. Despite these considerations, the present data, together with data from several other reports, markedly indicate that the inhibition of this pathway should be actively evaluated as a novel anticancer therapy. AMD3100 or other CXCR4-specific inhibitors should be developed and tested as therapies for human prostate cancer.

In conclusion, the present study showed that the CXCR4-specific antagonist, AMD3100, effectively inhibited SDF-1-induced CXCR4/Akt signal transduction in the PC-3 cells. Moreover, AMD3100 clearly suppressed tumor growth in the nude mice inoculated with the PC-3 cells, and AMD3100-treated PC-3 tumors showed lower levels of microvessel formation and a lower immunoreactivity for the proliferation marker Ki-67 and the anti-apoptotic marker Bcl-2 compared with control tumors *in vivo*. Thus, the study indicates that CXCR4 targeting may represent an effective strategy for the treatment of CRPC.
